# One-Dimensional Implantable Sensors for Accurately Monitoring Physiological and Biochemical Signals

**DOI:** 10.34133/research.0507

**Published:** 2024-10-16

**Authors:** Qianming Li, Wen Wang, Haotian Yin, Kuangyi Zou, Yiding Jiao, Ye Zhang

**Affiliations:** National Laboratory of Solid State Microstructures, Jiangsu Key Laboratory of Artificial Functional Materials, Chemistry and Biomedicine Innovation Center, Collaborative Innovation Center of Advanced Microstructures, College of Engineering and Applied Sciences, Nanjing University, Nanjing 210023, China.

## Abstract

In recent years, one-dimensional (1D) implantable sensors have received considerable attention and rapid development in the biomedical field due to their unique structural characteristics and high integration capability. These sensors can be implanted into the human body with minimal invasiveness, facilitating real-time and accurate monitoring of various physiological and pathological parameters. This review examines the latest advancements in 1D implantable sensors, focusing on the material design of sensors, device integration, implantation methods, and the construction of the stable sensor–tissue interface. Furthermore, a comprehensive overview is provided regarding the applications and future research directions for 1D implantable sensors with an ultimate aim to promote their utilization in personalized healthcare and precision medicine.

## Introduction

Implantable sensors, serving as front-end antennae for the collection of human health information [1–3], can monitor biochemical and physiological signals at the implant site in real time [4–7]. Due to their significant application value in human health monitoring, disease diagnosis, drug evaluation, and other fields, implantable sensors have made significant progress in recent years. Implantable sensors with a one-dimensional (1D) configuration provide a viable solution for long-term monitoring of signals in vivo due to their unique advantages. Firstly, the radial miniaturization of 1D sensors enables minimally invasive implantation by injection or catheter-assisted methods, thereby reducing the risk of major trauma surgery [8–10]. Secondly, as the dimensions of the material are reduced (for example, from metal blocks to metal foils to metal wires), the flexibility of the material in terms of bending stiffness is significantly decreased [11–13]. This allows the mechanical properties of the material to be well matched to soft tissues, thereby forming a compliant sensor–tissue interface and minimizing the foreign body reaction [14]. Thirdly, the 1D structure enables flexible sensor designs, including braiding, twisting, and spiraling, which allows for the potential for multifunctionality [15,16]. Finally, these 1D implantable sensors can be easily removed after the completion of the sensing task, thus avoiding the necessity for a second operation [17].

A series of 1D implantable sensors have been thus extensively explored in the past decades, and they are expected to greatly prompt the development of the next generation of implantable biomedical devices. For instance, surgical sutures for monitoring wounds, interface-stabilized fiber sensors for monitoring amniotic fluid, and fibrous, low-cost gastrointestinal manometers have been explored [18–20]. It is thus necessary to thoroughly review the development of 1D implantable sensors to identify the main research directions for the next step. In this review, compared to previously published reviews on implantable sensors, we underscored the advantages of 1D structures, particularly their distinctiveness in terms of implantation methods, integration, and applications. We first categorized 1D implantable sensors and summarized the materials used for various types of sensors. Emphasizing the structural features of 1D sensors, we then discussed the design strategies for these sensors, including assembly and integration methods, sensor–tissue interfaces, and implantation techniques. Finally, we presented the applications of 1D implantable sensors, highlighting their potential for advancing brain science research and enabling early disease detection and warning. We also addressed the challenges that remain and suggested areas for future exploration.

## 1D Implantable Sensors

1D implantable sensors are generally divided into 2 categories based on their application areas: sensors for physiological monitoring and sensors for biochemical detection.

### Physiological monitoring sensors

Physiological monitoring sensors were implanted into specific organs or attached to the surface of tissues, continuously and in real-time recording physiological parameters such as internal temperature, bioelectric changes, and mechanical changes [21–24]. These parameters provide important data for clinical diagnosis and postoperative management.

Temperature sensors monitor local tissue temperature changes in real time. These temperature variations can indicate conditions like inflammation and infection, serving as a reference for postoperative monitoring and health management [25–27]. For instance, postoperative infections and inflammation are common complications. Implanting a 1D temperature sensor allows real-time monitoring of temperature changes at the surgical site, aiding doctors in assessing the surgical outcome and recovery status.

Electrophysiological sensors include sensors for monitoring electromyography, electroencephalography, and electrocardiography [28–31]. These sensors provide crucial data for diagnosing neurological and cardiovascular diseases. For instance, the 1D electroencephalography sensor monitors abnormal brain discharges, offering early warnings for psychiatric patients [32].

Mechanical sensors are used to detect mechanical signals such as strain and pressure. These sensors are commonly used to monitor mechanical changes in tissues or organs, such as gastrointestinal pressure sensors, soft tissue strain sensors, and blood pressure sensors [33]. For example, gastrointestinal motility disorders can lead to digestive system diseases, including gastroesophageal reflux disease, gastroparesis, pseudo-obstruction, and chronic constipation. Assessing the tone and contractile patterns of the gastrointestinal tract with 1D pressure sensors is crucial for diagnosing gastrointestinal motility disorders [19].

### Biochemical monitoring sensors

In addition to physiological signals, it is essential to monitor health status at the molecular level. These sensors usually need to be in direct contact with the biochemical markers, so they are often implanted in environments such as blood, cerebrospinal fluid, interstitial fluid, ascites, amniotic fluid, and other body fluids [18,34–36]. These sensors can identify and quantify various biochemical molecules, providing critical information for early diagnosis, monitoring, and treatment of disease.

Metabolite sensors, which detect glucose, lactate, urea, and ascorbic acid, play a crucial role in managing diseases such as diabetes and metabolic syndrome [33,37–39]. For instance, implanted glucose sensors enable diabetic patients to monitor their blood glucose levels in real time, guiding insulin use [40]. Additionally, lactate concentration serves as an indicator of tissue hypoxia and exercise intensity [18,41].

Neurotransmitter sensors are used to detect neurotransmitters in body fluids such as dopamine, serotonin, glutamate, and acetylcholine [42–44]. These sensors play an important role in neuroscience research and the diagnosis of mental disorders. For example, 1D sensors for monitoring brain dopamine help diagnose conditions such as Parkinson’s disease and schizophrenia [45].

Ion sensors are used to detect various ions in body fluids, such as potassium ions, sodium ions, calcium ions, etc. Changes in ion concentration are often associated with various diseases [46]. For example, monitoring the concentration of potassium ions in the blood can assess cardiovascular diseases such as arrhythmias, while the concentration of calcium ions is closely related to bone health.

Gas sensors that detect the concentration of specific gases in the body, such as nitric oxide (NO) [47], hydrogen sulfide [48], and oxygen sensors [49], play a critical role in monitoring and research, particularly in the cardiovascular, neurological, and immune systems [50]. For example, NO plays a key role in vasodilation and blood pressure regulation, and NO sensors can help assess blood flow and cardiovascular health.

## Materials of 1D Implantable Sensors

Flexible conductive materials are key components in implantable sensors, which are mainly divided into 3 categories: (a) hydrogel-based materials, (b) liquid metal-based materials, and (c) carbon-based materials.

### Hydrogel-based materials

Hydrogels are cross-linked polymer networks with high water content [51]. Structurally, they resemble the extracellular matrix of natural tissues and have Young’s modulus similar to that of soft tissues, typically ranging from 1 Pa to 1 MPa [52,53]. This similarity reduces mechanical stimulation and damage to surrounding tissues, demonstrating excellent biocompatibility.

**Fig. 1.**
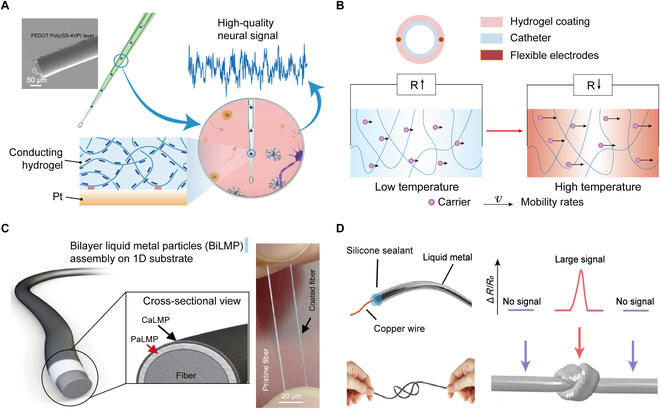
Design and fabrication of 1D implantable sensors for physiological monitoring. (A) Schematic diagram and scanning electron microscopy (SEM) image of a neural electrode modified with conductive hydrogel, which imparts biocompatibility and mechanical properties to the neural electrode, thereby enhancing the efficiency of electrical stimulation and recording. Reproduced with permission [58]. Copyright, 2023, Wiley-VCH. (B) Schematic diagram of the structure of a real-time temperature sensor for monitoring surgical sites based on ion-conductive hydrogel with temperature sensitivity, and the mechanism of temperature sensitivity. Reproduced with permission [25]. Copyright, 2023, Wiley-VCH. (C) Schematic diagram and photograph of a highly conductive multilayer-structured liquid metal composite fiber electrode. The BiLMP coating consists of 2 layers: PaLMP (poly (styrene sulfonate)-linked liquid metal particles [LMP]) and CaLMP (CNT-linked LMP). Reproduced with permission [62]. Copyright, 2023, Springer Nature. (D) Schematic diagram of the preparation and pressure response mechanism of a pressure sensor for gastrointestinal pressure measurement developed based on the piezoresistive effect of liquid metal. When pressure is applied to the junction, a change in resistance is detected. Reproduced with permission [19]. Copyright, 2022, Springer Nature.

Hydrogels with electronic conductivity can be obtained by constructing conducting polymer hydrogels or adding conductive fillers (such as metallic nanomaterials and carbon-based nanomaterials) to the hydrogels [54]. The former is widely used in implantable sensors because of its better biocompatibility. Conducting polymer hydrogels typically consist of intrinsically conducting polymers with π-π conjugated structures as networks such as poly (3,4-ethylenedioxythiophene):poly (styrene sulfonic acid) (PEDOT:PSS), polypyrrole, and polyaniline [55,56]. Among them, PEDOT:PSS hydrogels show high electrical conductivity. At the same time, the charge storage capacity and charge injection capacity, 2 important parameters for bioelectronic applications, reach 60 and 8.3 mC cm^−2^, respectively, meeting the performance requirements of brain–computer interface electrophysiological sensor [57]. For example, fiber neural electrodes can be prepared by electrochemically polymerizing poly(3,4-ethylenedioxythiophene):poly (styrene sulfonate-*co*-4-vinyl pyridine) hydrogel coatings on platinum wires (Fig. 1A). These electrode surfaces matched the modulus of brain tissue (~1 kPa), which helped to reduce inflammatory responses. Additionally, they maintain impedance values below 250 kΩ (1 kHz) after 4 weeks of operation in mouse brains, enabling high-resolution electrophysiological recordings [58].

Hydrogels with ionic conductivity were obtained by adding conductive ionic salts (such as NaCl and LiCl) to hydrogels (e.g., polyacrylic acid, polyacrylamide, polyvinyl alcohol, alginate, chitosan, etc.) [59,60]. The internal structure and charge carrier migration rate of conductive hydrogels vary with temperature, thereby changing their ionic conductivity. Based on their temperature sensitivity, composite conductive hydrogels can be used as temperature sensors. For example, a temperature sensor designed for monitoring local wound infection was developed by modifying a medical catheter with a polyacrylamide/polyacrylic acid/chitosan conductive hydrogel coating, using Na^+^ and Cl^−^ as charge carriers (Fig. 1B) [25]. This sensor exhibited a high-temperature coefficient of resistance of up to 2.90% °C^−1^ and a resolution of 0.1 °C, making it capable of detecting a 1 °C abnormal change in local tissue within 1 min. The highly sensitive temperature sensor was particularly important for real-time monitoring of postoperative wound infection.

### Liquid metal-based materials

Liquid metals, represented by gallium-indium alloys, have excellent electrical conductivity (3.4 × 10^6^ S m^−1^) and biocompatibility, which makes them ideal materials for accurately monitoring and conducting electrical signals in vivo [61]. Due to their fluidity, liquid metals were further fixed for 1D flexible electrodes (e.g., by compounding them with polymers to form a 1D configuration or encapsulating them with an elastomer tube). For example, 1D neural electrodes prepared with poly (styrene sulfonate)-modified liquid metal exhibited high conductivity up to 2.24 × 10^6^ S m^−1^ (Fig. 1C), and the conductivity remained stable under stretching conditions (~20% strain). After being implanted in the cerebral cortex for 11 weeks, the monitored brain electrical signals still maintained a low noise level (~10 signal-to-noise ratio) [62].

Furthermore, the ability of liquid metals to change shape and volume under external force can be used to construct pressure sensors. Specifically, liquid metal is encapsulated in a silicone tube to form channels of specific shapes. When external pressure is applied, the length of the channels increases and the cross-sectional area decreases, increasing the overall resistance of the material, which enables a response to pressure changes. As shown in Fig. 1D, a pressure sensor for gastrointestinal pressure measurement was fabricated by injecting liquid metal into a medical catheter and sealing both ends. When it was implanted in the gastrointestinal tract, it monitored small pressures within 50 kPa while exhibiting high linearity (*R*^2^ > 0.985) and low-hysteresis response (<0.5%) [19].

### Carbon-based materials

Carbon materials such as carbon nanotubes (CNTs), graphene, and carbon fiber have been widely explored to develop implantable sensors due to their high electrochemical activity and electrocatalytic properties [63].

**Fig. 2.**
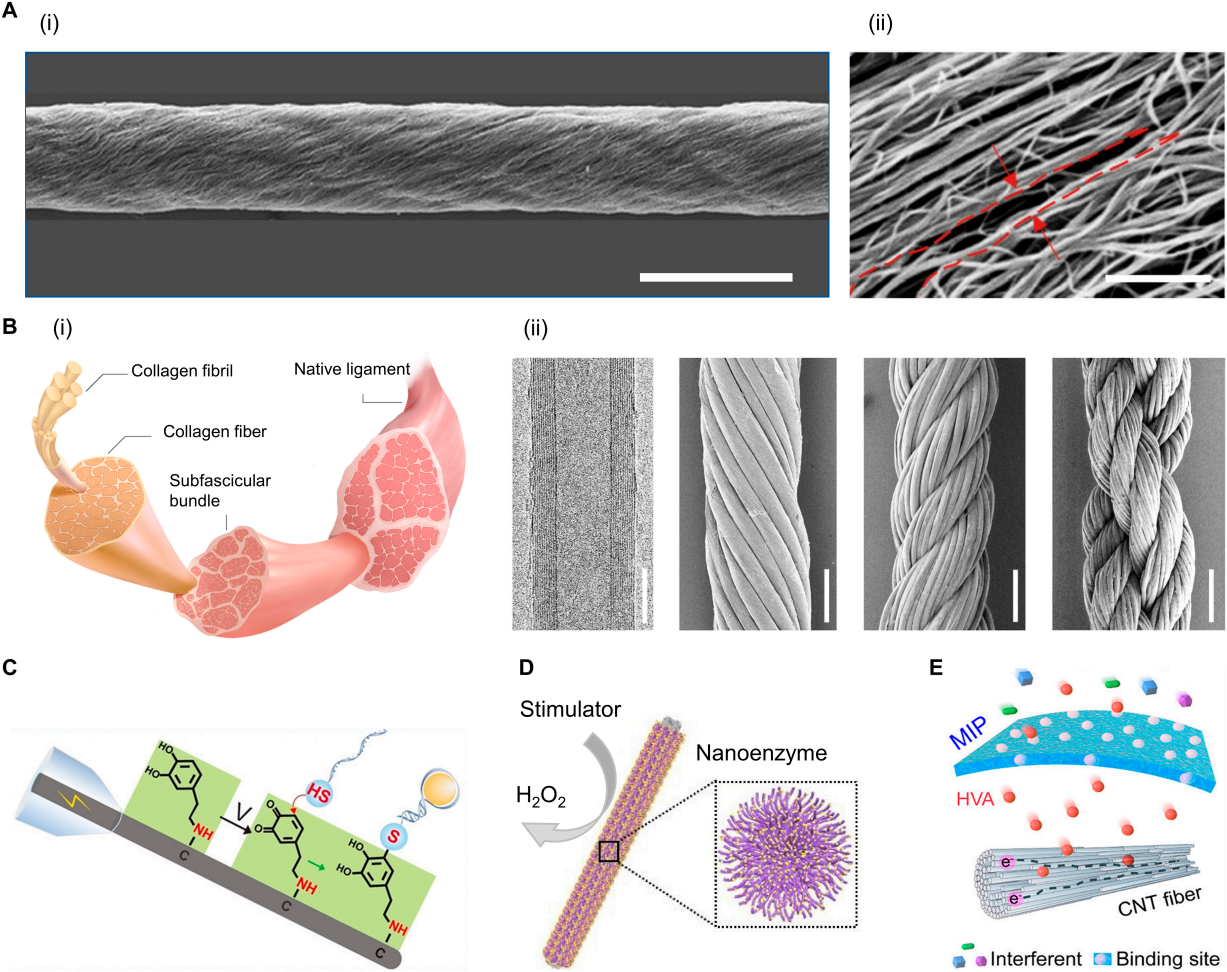
Design and fabrication of 1D implantable sensors for biochemical signal monitoring. (A) SEM images of a single CNT fiber prepared by dry spinning (i) (scale bar, 20 μm) and a magnified view of a primary CNT fiber showing nanoscale gaps between adjacent CNTs indicated by red lines (ii) (scale bar, 500 nm). Reproduced with permission [16]. Copyright, 2017, Springer Nature. (B) Schematic diagram of the multilevel structure of a ligament (i). From left to right, the multilevel CNT fiber of the bionic ligament (ii), transmission electron microscope image of multiwalled CNTs (scale bar, 5 nm), SEM image of secondary CNT fiber obtained by axially helically arranging 30 primary CNT fibers (scale bar, 40 μm), SEM image of tertiary fiber twisted from 4 secondary fibers (scale bar, 80 μm), and SEM image of double-helix quaternary fiber prepared from 2 tertiary fibers (scale bar, 150 μm). Reproduced with permission [65]. Copyright, 2023, Springer Nature. (C) Preparation of a high-sensitivity dopamine sensor by grafting aptamers on the surface of the fiber electrode. Reproduced with permission [67]. Copyright, 2022, Wiley-VCH. (D) Preparation of a hydrogen peroxide sensor by electrochemically depositing nano-manganese dioxide on the surface of the CNT fiber. Reproduced with permission [68]. Copyright, 2020, Elsevier. (E) Preparation of a highly selective HVA sensor by electrochemically depositing molecularly imprinted polymers (MIPs) on the surface of the fiber electrode. Reproduced with permission [34]. Copyright, 2024, American Chemical Society.

The electrical, mechanical, and thermal properties of individual CNTs and their 1D structure endow CNTs with great potential in 1D implantable sensors [64]. Through dry spinning processes, CNTs can be further assembled into macroscopic CNT fibers by leveraging the entanglement and van der Waals forces between adjacent CNTs (Fig. 2A) [16]. In addition to retaining the original properties of CNTs, CNT fibers also possess a larger specific surface area, approximately 500 to 850 m^2^ g^−1^ [34,47]. By mimicking the layered and helical structures of natural soft tissues, CNT fibers can be further twisted to prepare multilevel helical fibers (Fig. 2B). These fibers exhibit muscle-like bending stiffness (~10^−8^ nN m^2^) and demonstrate good mechanical compatibility with soft tissues when implanted [65].

Due to their excellent electrochemical activity and specific surface area, CNT fibers are commonly used to fabricate 1D implantable biochemical sensors. Depending on the target molecules detected and the application scenarios, various immobilization methods such as physical adsorption, chemical covalent bonding, cross-linking, encapsulation, and electropolymerization can be selected to immobilize biorecognition elements (such as enzymes, antibodies, and aptamers) on the surface of CNT fibers [66]. The biorecognition elements specifically bind to target molecules and are further converted into measurable electrical signals to design biochemical sensors. As shown in Fig. 2C, aptamers were covalently coupled to the fiber electrodes, resulting in a dopamine sensor with excellent selectivity and sensitivity, a detection limit as low as 13 nM, and a sensitivity of 4 nA μM^−1^, providing a powerful tool for continuous monitoring of dopamine in the brain of living animals [67].

Although natural biomimetic elements can endow biochemical sensors with good selectivity and sensitivity, they sometimes sacrifice stability. For example, enzymes or antibodies are easily affected by environmental factors, such as mechanical deformation, pH, temperature, etc., which in turn lose or reduce their activity and sensor function. A promising direction here is to achieve stable binding with biomarkers through artificially prepared recognition elements. For instance, the electrochemical deposition of nanozymes (i.e., artificially prepared inorganic nanomaterials) on the surface of carbon fibers achieved specific recognition of biochemical substances (Fig. 2D) [68]. Additionally, molecularly imprinted polymers, known as “artificial receptors”, allowed only molecules that precisely matched the binding sites to pass through, which might have been one of the future development directions for biochemical sensors. As shown in Fig. 2E, an implantable electrochemical fiber sensor was developed by electrochemically depositing molecularly imprinted polymers on the surface of CNT fibers for real-time monitoring of homovanillic acid (HVA) molecules in blood. This sensor’s response to HVA was 12.6 times higher than that of similar metabolites and exhibited selectivity with an accuracy of 97.8%, and the electrochemical performance of the sensor remained stable after 4 weeks of implantation [34].

## Design Strategy of 1D Implantable Sensors

The application of the obtained 1D sensors in vivo is challenging. First, in vivo space is limited, which requires sensors to be as integrated as possible to reduce tissue damage. Second, long-term monitoring of the sensor in vivo requires a compliant interface between the sensor and the surrounding tissue to reduce foreign body reactions and maintain sensor performance. Finally, the implantation process also needs to be as minimally invasive as possible to reduce the risk of infection associated with implantation [69,70]. Here, we will discuss how to achieve highly integrated, stable sensor–tissue interfaces and minimally invasive implantation methods for 1D implantable sensors. Moreover, comparison between 1D implantable sensor and existing technologies was made to validate its advancement (Table).

**Table.** Comparison of 1D implantable sensors with existing technologies

Sensor type	Sense signal	Ref.	Implantation method	Size	Electrode material	Young’s modulus	Long-term stability
**1D sensor**	Pressure	[19]	Insertion	Diameter ~0.64 mm	EGaIn /silicone	/	2 h
	Temperature	[25]	Insertion	Thicknesses~ 100 μm	CNT fiber	~4 kPa	7 d
	Bioelectricity	[79]	Puncture	Diameter ~20 μm	CNT fiber	~10 kPa	4 wk
		[92]	Puncture	Diameter ~15 μm	PEDOT:PSS	300 kPa	5 mo
	Glucose	[18]	Injection	Diameter ~50 μm	CNT fiber	8 MPa	7 d
		[38]	Injection	Diameter ~150 μm	CNT fiber	/	7 d
	Lactate	[18]	Injection	Diameter ~50 μm	CNT fiber	8 MPa	7 d
	DA	[32]	Bore hole	Diameter ~100 μm	CNT fiber	~100 Kpa	8 wk
		[67]	Bore hole	Diameter ~10 μm	Carbon fiber	/	120 h
		[22]	Bore hole	Diameter ~7 μm	Carbon fiber	/	21 d
	NO	[47]	Bore hole	Diameter ~50 μm	CNT fiber	0.64 Mpa	4 wk
	K^+^	[80]	Bore hole	Diameter ~60 μm	CNT fiber	65 Mpa	6 mo
	pH	[78]	Puncture	Diameter <300 μm	EGaIn /SBS	13 Mpa	24 h
		[49]	Bore hole	Diameter ~10 μm	Carbon fiber	/	1 h
**2D/3D sensors**	Temperature	[93]	Surgery	0.5 × 0.5 mm^2^	Au	<70 kPa	3 wk
		[94]	Surgery	12.8 × 8.2 × 5.8 mm^3^	/	/	~5 mo
	Bioelectricity	[95]	Surgery	4 × 4 mm^2^	Hydrogel	90 kPa	4 wk
	Strain	[96]	Surgery	1 × 2 cm^2^	Au-TiO_2_ NWs	647 kPa	48 h
		[97]	Surgery	~1 × 1 cm^2^	Graphene	/	~4 h
	Glucose	[98]	Surgery	~3 × 10 mm^2^	Hydrogel	/	45 d
	Lactate	[99]	Surgery	4 × 4 × 15 mm^3^	Copper	/	144 h
	DA	[100]	Surgery	12 × 8.5 × 3.2 mm^3^	Au	/	5 d
	K^+^	[101]	Surgery	4.2 × 1.7 mm^2^	CNT	1.2 MPa	2 wk
	pH	[102]	Surgery	0.75 × 0.75 mm^2^	Silicon	/	5,000 s
		[103]	Surgery	1 × 2 cm^2^	Silicon oxide	/	114 h
	NO	[104]	Surgery	1 × 1 cm^2^	Au nanomembrane	/	5 d

### Assembly integration of 1D sensors

Due to the high flexibility and structural advantages of 1D sensors, they can achieve high temporal and spatial resolution detection of complex internal signals through simple processes such as knotting, winding, twisting, and torsion [16].

**Fig. 3.**
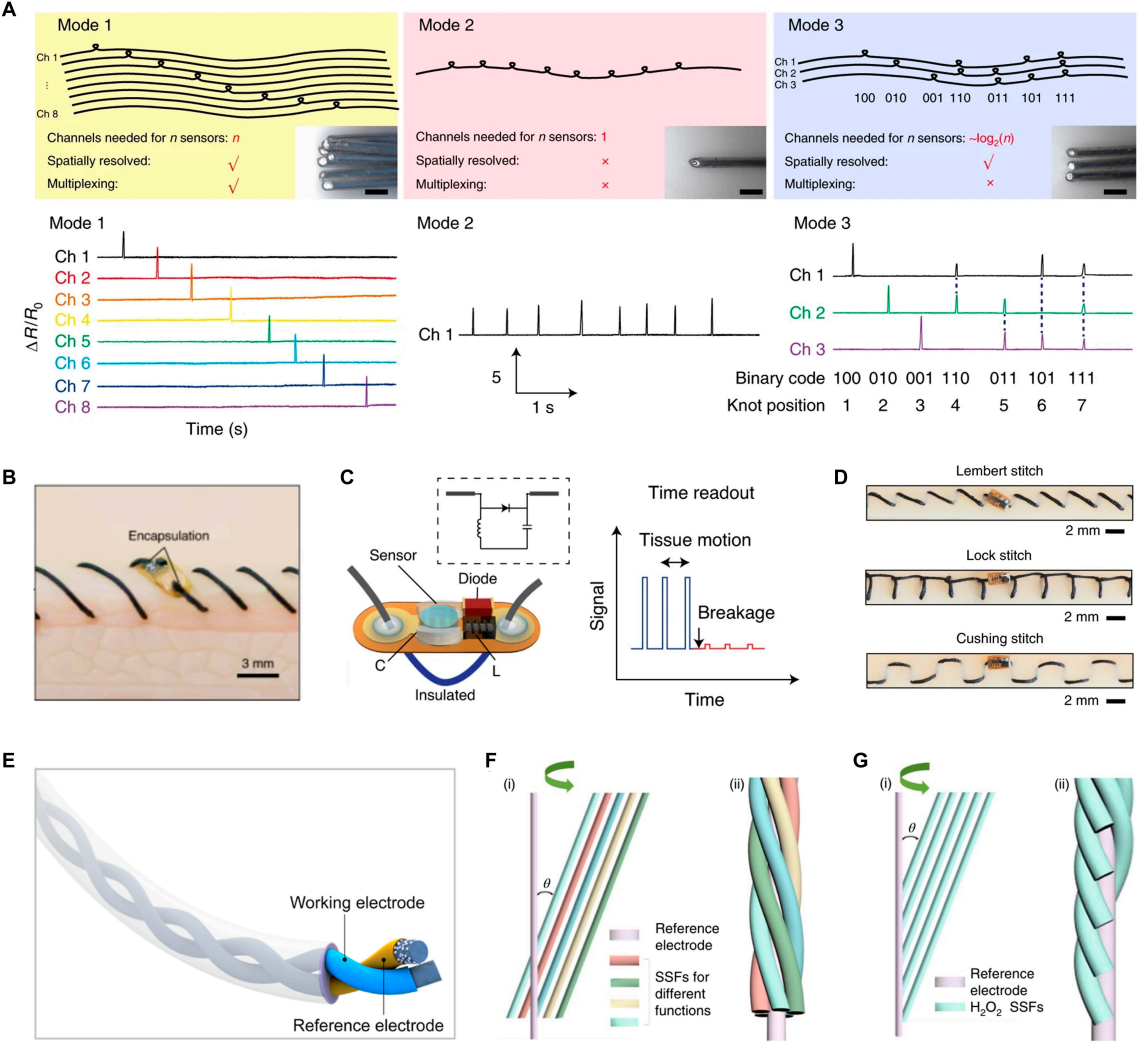
Assembly integration of 1D sensors. (A) Schematics of modes 1, 2, and 3 of multiplexing (top). Inset: Optical images of the total device cross-section of each mode. Scale bars, 1 mm. Multichannel resistance recording of each mode from the rolling test (bottom). Reproduced with permission [19]. Copyright, 2022, Springer Nature. (B) The electronic suture for tissue stitching. (C) Schematic and equivalent electrical circuit of the electronic suture (left). The module incorporates a Schottky diode, a capacitive sensor, and a tuning inductor, while the suture functions as a dipole antenna. Wireless readout of the sutures in the time-resolved mode (right). Changes in the electrical length of the suture result in temporal variations in the backscattered harmonic signal. (D) Three types of patterns of electronic sutures inside the tissue. (B to D) Reproduced with permission [20]. Copyright, 2021, Springer Nature. (E) Schematic of a fiber electrochemical sensor consisting of a working electrode and a reference electrode. Reproduced with permission [34]. Copyright, 2024, American Chemical Society. (F) Schematic of the preparation process (left) and schematic of the structure (right) of a fiber electrochemical sensor with radial terminals for monitoring of multiple biochemicals. (G) Schematic of the preparation process (left) and schematic of the structure (right) of a fiber electrochemical sensor with axial terminals and 5 evenly spaced sensing fibers for spatial analysis. (F and G) Reproduced with permission [15]. Copyright, 2020, Springer Nature.

For example, different gastrointestinal monitoring needs could be addressed by designing the number and positions of nodes in 1D sensors (Fig. 3A) [19]. In mode 1, each node occupied one channel, which was suitable for simultaneous measurements at different locations and achieved maximum spatial resolution, but it had the disadvantage of being bulky. Mode 2 tied multiple nodes on a single tube, which was most economical for data recording but could not spatially resolve signals if they occurred simultaneously. Mode 3 used combinations of different nodes and decoupled the signals to resolve temporally overlapping pressure responses, balancing spatial resolution and device complexity. Additionally, by winding the 1D sensor multiple times within the tissue (Fig. 3B) [20], it could not only act as a suture but also function as a dipole antenna, providing wireless response capability by backscattering incident signals, enabling the monitoring of deep surgical tissue sites (Fig. 3C). Different suture patterns could also achieve monitoring of tissues at different depths (Fig. 3D).

Electrochemical methods are the most common way to detect biochemical substances in vivo. Typically, an electrochemical sensor requires at least one working electrode and a reference electrode to form a circuit. Due to the unique 1D flexibility of fibers, a composite fiber electrochemical sensor was obtained by twisting the working fiber and the reference fiber together, which enabled the monitoring of a specific biochemical marker (Fig. 3E) [34]. For simultaneous monitoring of multiple biochemicals, several fiber electrochemical sensors were helically assembled to obtain an integrated fiber electrochemical sensor (Fig. 3F) [15]. Additionally, multiple fiber electrochemical sensors were further twisted together along the axial direction at regular intervals, allowing for spatial distribution detection of target analytes, with spatial resolution adjustable from micrometers (equivalent to the diameter of the fiber) to millimeters (Fig. 3G). For example, twisting 5 hydrogen peroxide fiber electrochemical sensors with 1 Ag/AgCl reference electrode produced an integrated electrochemical sensor with axial interval distribution [15]. When implanted into the solid tumor of a nude mouse, it achieved real-time analysis of the chemical distribution within the tumor at different growth stages, which was valuable for understanding tumor occurrence, development, and survival in hypoxia. Fiber electrochemical sensors provide timely and accurate information, offering a promising platform for future real-time health monitoring affordably and comfortably.

### Sensor–tissue interface

Long-term continuous monitoring in vivo requires a stable and robust sensor–tissue interface. Poor sensor–tissue interfaces may trigger strong rejection reactions, resulting in biofilm formation on the sensor surface, reducing effective contact between the sensor and tissue, and thereby diminishing the sensor’s sensitivity and selectivity [71]. Additionally, poor sensor–tissue interfaces can activate the body’s immune system, causing intense inflammatory reactions and complex interactions between molecular and cellular components, which interfere with the monitoring environment and lead to inaccurate sensor results [58]. Thanks to the minimal invasiveness and high mechanical flexibility of 1D implantable sensors, they can better adapt to the morphology of tissues, reduce mechanical damage and inflammatory reactions to the surrounding tissues, and have outstanding advantages in forming a stable sensor–tissue interface.

**Fig. 4.**
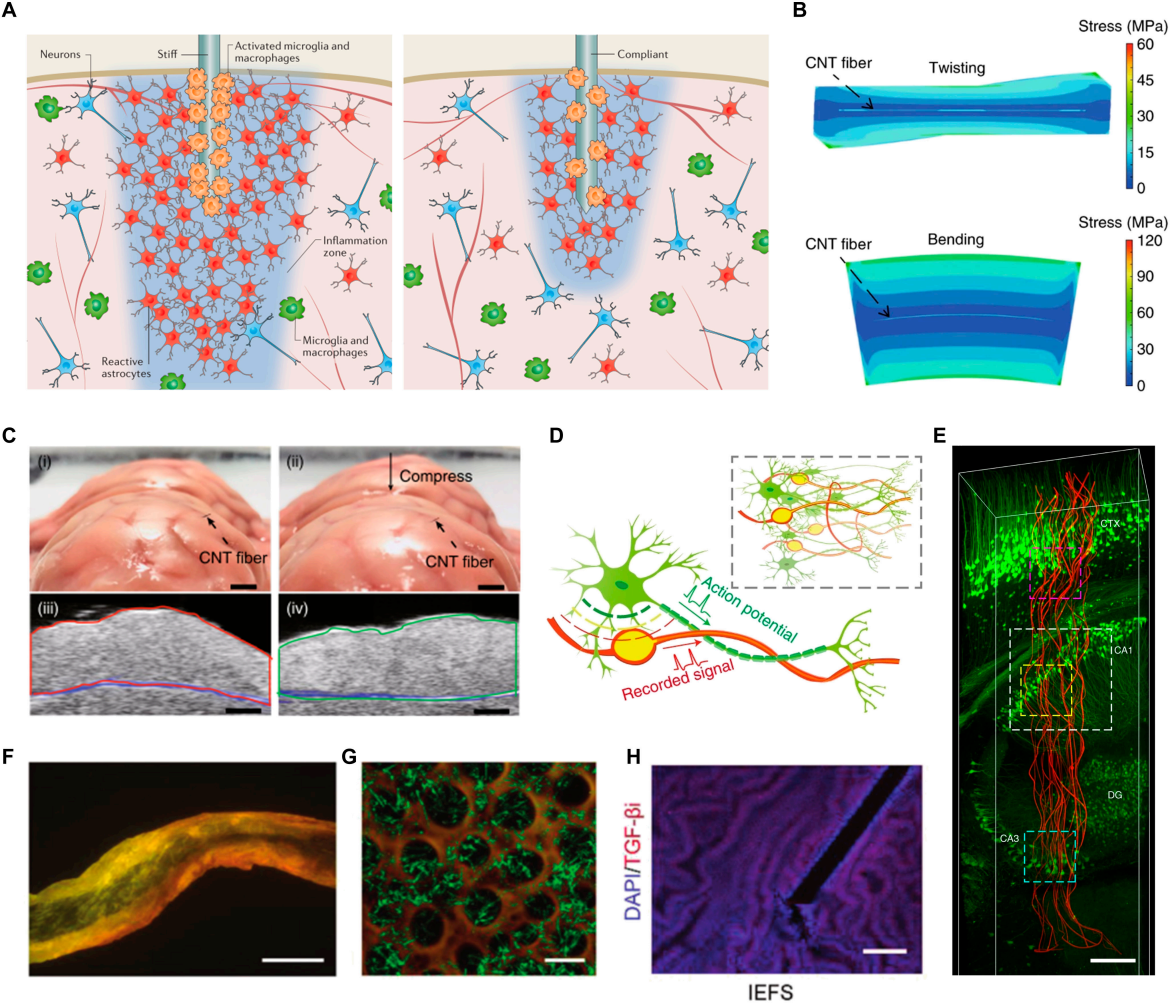
Sensor–tissue interface. (A) Schematic of foreign body reaction around a rigid implant and a soft implant. Reproduced with permission [73]. Copyright, 2016, Springer Nature. (B) The stress simulation of the CNT fiber in a tissue-like medium under twisting (top) and bending (bottom) shows a very low level of stress with a uniform distribution around the fiber. (C) Optical images (i and ii) and the corresponding photoacoustic images (iii and iv) of a brain, which was implanted with a CNT fiber, from a pig before (i and iii) and after (ii and iv) compression. Scale bars, 1 cm. (B and C) Reproduced with permission [15]. Copyright, 2020, Springer Nature. (D) Schematics showing the structural similarity between a neural probe (NeuE) and neurons from the subcellular level to the network level (inset). (E) 3D reconstructed interface between neurons (green) and NeuE (red) at 6 weeks postimplantation. Scale bar, 200 μm. (D and E) Reproduced with permission [75]. Copyright, 2019, Springer Nature. (F) Fluorescence micrograph of the electrochemical fiber sensor composed of a sensing fiber core (black) and a restorative gel sheath (yellow). Scale bar, 100 μm. (G) Fluorescence micrograph of the restorative gel composed of sericin (brown) and collagen (green). Scale bar, 10 μm. (H) Immunofluorescence staining for transforming growth factor-βi (TGF-βi) (red) and 4′,6-diamidino-2-phenylindole (DAPI) (blue) in the amnion after implantation of the electrochemical fiber sensor for 3 d. Scale bar, 100 μm. (F to H) Reproduced with permission [18]. Copyright, 2024, Wiley-VCH GmbH.

Various methods have been used to improve the sensor–tissue interface, such as optimizing the mechanical properties and microstructure of the materials. The flexibility of the implanted sensor affects the inflammation and healing process at the implantation site, as rigid implanted sensors cannot accommodate the natural micro/macro movements of the tissue [72]. Existing studies have demonstrated that mechanical differences between the tissue and the implanted material could create adverse strain fields near the device, potentially leading to irreversible tissue damage and sensor failure (Fig. 4A) [73]. To adapt to the mechanics of the tissue, the Young’s modulus of the implanted sensor is a critical design parameter. The Young’s modulus of human tissues varies significantly depending on the tissue type. The following are the typical ranges of Young’s modulus for some common human tissues: brain tissue is about 0.5 to 10 kPa, membranous tissues (such as pleura and peritoneum) are about 10 to 100 kPa, intestinal tissues are about 1 to 500 kPa, soft tissues (such as skin, muscle, and fat) are about 10 to 1,000 kPa, and cartilage is about 0.3 to 2 MPa [14,73,74]. A simple method is to use flexible materials to manufacture the sensors, making them soft and stretchable to match the mechanics of the implantation site. As shown in Fig. 4B, by using multilevel twisted CNT fibers, the fiber sensor had Young’s modulus of the same order of magnitude as the implanted tissue (approximately megapascal level), allowing the sensor and tissue to conformally change together (Fig. 4C) [15]. One month after implantation, the tissue at the implantation site did not exhibit inflammation, and the sensor functioned normally.

Another factor affecting the sensor–tissue interface is the size of the implanted sensor. When the size and morphology of the implanted sensor are similar to the microstructure of the implantation site, it facilitates cell migration at the implantation site and promotes tissue repair. As shown in Fig. 4D, a neural probe was designed to mimic the subcellular structural features and mechanical properties of neurons. These subcellular structural features were proven to promote the migration of endogenous neural cells, allowing the device and neurons to interpenetrate and form a stable interface seamlessly integrated with the brain (Fig. 4E) [75]. This achieved stable recording of individual cells in a physiological environment without quality loss for 3 months, opening opportunities for the next generation of brain–computer interfaces.

In addition to the biomimetic design of the overall device structure, the surface of the implanted sensor can be modified to enhance the stability of the sensor–tissue interface. As shown in Fig. 4F and G, a micrometer-scale porous hydrogel was prepared on the surface of the fiber sensor. The hydrogel, similar to the extracellular matrix in structure and composition, promoted cell migration and differentiation, ultimately enabling seamless integration of the sensor into the tissue and achieving long-term stable monitoring in dynamic environments (Fig. 4H) [18].

### Effective implanting methods

**Fig. 5.**
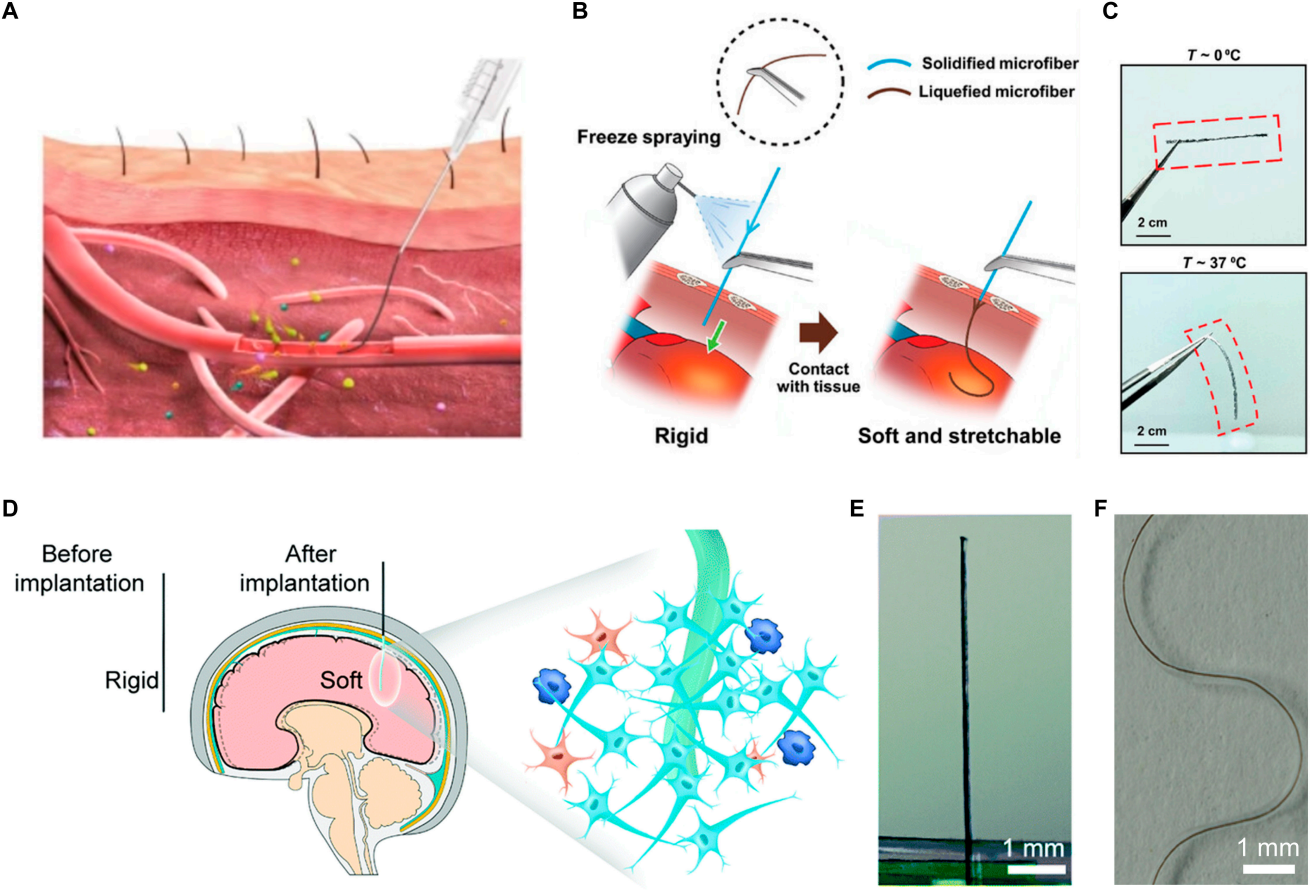
Methods for the implantation of 1D sensors. (A) Schematic of a fiber sensor injected into a blood vessel with the assistance of a syringe. Reproduced with permission [75]. Copyright, 2020, Springer Nature. (B) The soft microfiber is frozen to be rigid, like a needle, by freeze-spraying. The solidified microfiber can penetrate tissues, and it recovers its original softness in response to the body temperature after implantation. (C) Optical images of the solidified microfiber at 0 °C and the liquefied microfiber at 37 °C. (B and C) Reproduced with permission [78]. Copyright, 2024, Wiley-VCH. (D) Microfiber neural probes with alterable elastic moduli ensure direct implantation and become soft after implantation, which reduces neuroinflammatory response. Photograph of the dry probes (E), and the wet probes (F) in artificial brain cerebrospinal fluid. (D to F) Reproduced with permission [79]. Copyright, 2020, Royal Society of Chemistry.

The 1D implantable sensor exhibits a high aspect ratio, excellent mechanical compliance, lightweight construction, and flexibility. It provides a smaller volume, cross-sectional area, and enhanced flexibility compared to planar sensors. Therefore, they are easier to insert deeply into tissues or natural openings using minimally invasive methods, significantly reducing surgical trauma and the risk of biomedical complications [76,77]. For example, fine CNT fibers with a 1D structure could better conform to the needle of a syringe, allowing one end of the fiber to be precisely injected into the target tissue while the other end remained outside the tissue for external connection (Fig. 5A) [15].

Besides implantation with the help of a syringe, 1D sensors can also achieve simplified and targeted implantation through variable elastic modulus. Initially, the rigidity of the sensor ensures direct and targeted insertion. After implantation, the device softens under physiological conditions and contacts the surrounding tissue compliantly. As shown in Fig. 5B, based on the phase transition characteristics of liquid metal (eutectic gallium-indium; melting temperature of about 15.5 °C), it penetrated heart tissue as sharply as a steel needle in its frozen state. After implantation, it regained its original softness due to the solid–liquid phase transition, with mechanical softness comparable to the target tissue (Fig. 5C) [78]. However, the low-temperature sensor insertion may affect the biological activity of surrounding cells. Additionally, fiber sensors that changed their elastic modulus by switching between dry and wet states were explored (Fig. 5D). The modulus of the dry fiber device was as hard as a metal wire, while the fiber sensor’s modulus approached that of brain tissue when wetted by body fluids after implantation (Fig. 5E and F) [79]. In summary, modulus-responsive 1D sensors are a promising implantation method, enabling low trauma and stable device–tissue interfaces.

## Application of 1D Implantable Sensors

### Applications in brain science research

Monitoring physiological and biochemical signals in the brain is critical to elucidating the causes of neurological disorders and understanding human emotions, behaviors, and cognitive processes [80–83]. 1D sensors can be implanted deep into the brain for extended periods of time while minimizing compression or damage to nerves, compared to 2D and 3D sensors. Additionally, it offers precise localization and monitoring of specific brain regions, allowing for high-spatial-resolution observation.

**Fig. 6.**
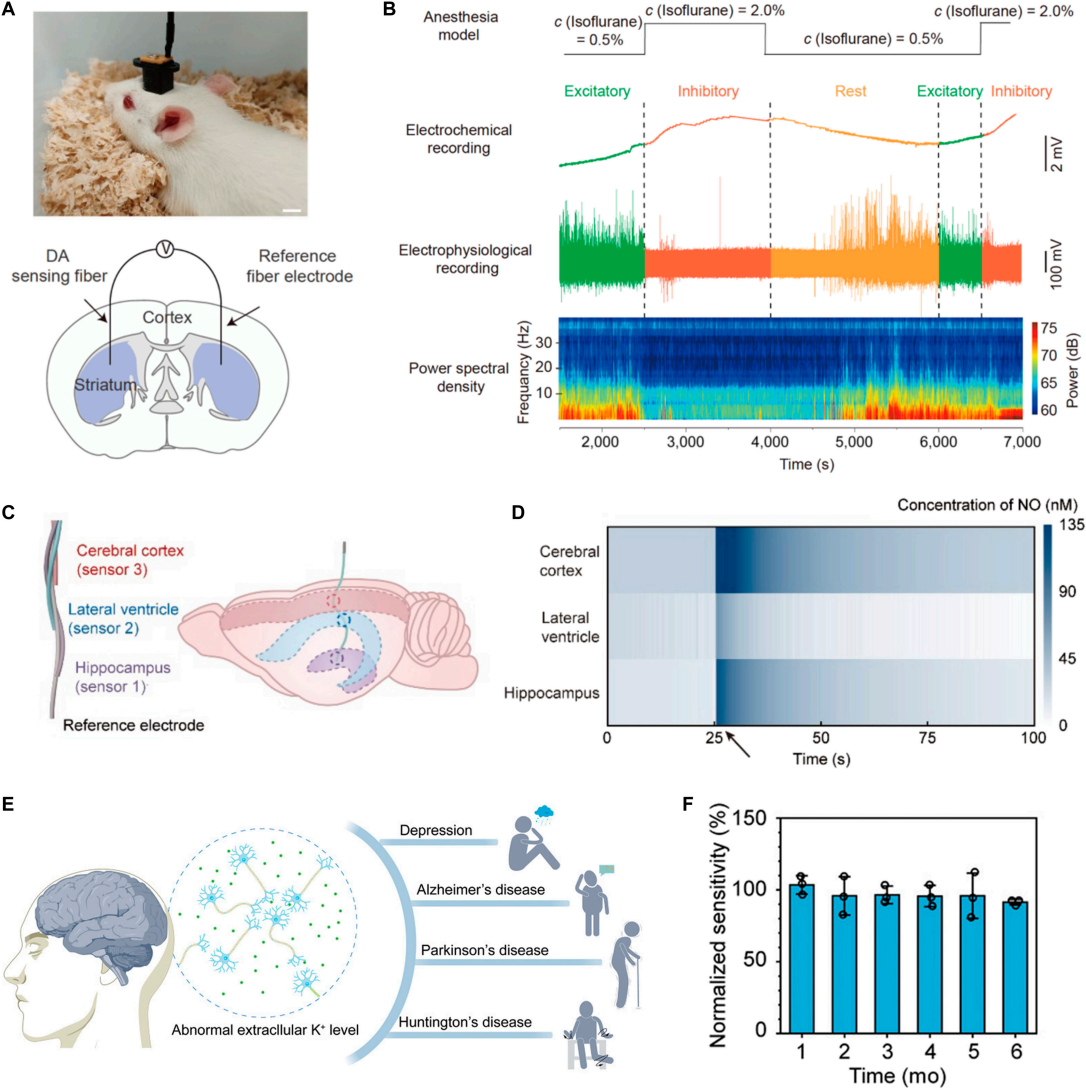
The application of 1D implantable sensors in brain science research. (A) Photograph of a rat after implantation of the CNT fiber-based sensor (top). Scale bar, 1 cm. Schematic illustration of the implantation position of a dopamine-sensing fiber and Ag/AgCl reference fiber (bottom). (B) Simultaneous recording of dopamine and electrophysiology by fiber sensor and fiber neural electrode during anesthesia of a rat and corresponding power spectral density were further calculated. (A and B) Reproduced with permission [32]. Copyright, 2021, Science China Press and Springer-VGH. (C) Schematic of the structure of a multi-ply NO sensing fiber constructed by twisting 3 CNT fiber-based NO sensors and an Ag/AgCl reference fiber together. The spatial analysis of different brain regions can be realized simultaneously by controlling the location of sensing sites. (D) Mapping of the spatial and temporal distribution of NO in 3 different regions of the multi-ply NO sensing fiber implanted in the rat brain during severe stroke. The arrows indicate the onset of severe stroke. (C and D) Reproduced with permission [47]. Copyright, 2024, Science China Material and Springer-VGH. (E) Conceptual illustration of abnormal extracellular K^+^ levels in the brain and the related chronic neuropsychiatric diseases to demonstrate the requirement of long-term monitoring of K^+^ levels in time. (F) Sensitivities of the sensor kept in artificial cerebrospinal fluid for 6 months, exhibiting long-term stability (*n* = 3, mean ± SD). (E and F) Reproduced with permission [80]. Copyright, 2024, Wiley-VCH.

For example, after implanting the dopamine-sensing fiber into the rat striatum (Fig. 6A), the sensor exhibited strong anti-interference properties and neuronal compatibility [32]. It also worked with an electrophysiological fiber sensor to simultaneously monitor changes in dopamine and electrical signals in the brain (Fig. 6B). In addition to monitoring individual brain regions, simultaneous monitoring of brain regions at different depths can be achieved by twisting the fiber sensor (Fig. 6C) [47]. As shown in Fig. 6D, the integrated fiber sensor allowed simultaneous monitoring of NO in the cerebral cortex, lateral ventricles, and hippocampus. With continuous improvements in sensor materials and design, fiber sensors targeting various biochemicals in the brain (e.g., hydrogen peroxide [38], glucose [15], ascorbic acid [45,49], and glutamate [84]) were developed. These advances in fiber sensors provided reliable tools for ongoing brain science research.

Potassium ions (K^+^) in the brain play a crucial role in mediating neural activity and promoting brain function. Dysfunction of K^+^ channels in cell membranes disrupts K^+^ transport, leading to abnormal extracellular K^+^ levels. These abnormalities are closely associated with a range of neurological disorders such as Parkinson’s disease, Alzheimer’s disease, and depression (Fig. 6E) [80]. However, existing assays do not allow long-term monitoring of the brain’s K^+^. As shown in Fig. 6F, a fiber electrochemical K^+^ sensor was designed for stable monitoring of K^+^ in the brain for up to 6 months. Applying it to depressed mice, the consistency between K^+^ fluctuations and behavioral traits was verified for the first time. This marks significant progress in unraveling the pathogenesis of neuropsychiatric disorders and evaluating the therapeutic effects of new drugs.

In addition to directly detecting biochemicals in the brain, fiber sensors can also be implanted into blood vessels to indirectly monitor biomarkers related to the brain with less invasiveness. HVA, a major metabolite of dopamine, crosses the blood–brain barrier and enters the circulatory system. Therefore, fluctuations of HVA in the blood are closely related to dopamine activity in the central nervous system. A highly selective implantable HVA fiber sensor based on molecularly imprinted polymers has been developed [34]. After a minimally invasive injection of this sensor into a rat vein, it successfully monitored parallel changes in blood HVA concentration induced by fluctuations in central nervous system dopamine and simultaneously showed excitatory behavior in rats.

### Early warning and diagnosis of diseases

1D implantable sensors are crucial for the early detection and diagnosis of major diseases such as diabetes [40], pregnancy disorders [18], and wound infections [25]. By in situ real-time monitoring of relevant markers through sensors, an early warning is issued when abnormal fluctuations occur, to timely intervene in the early stage of the disease and improve the cure rate of patients. For example, commercialized 1D implantable glucose sensors are already playing an important role in patients with diabetes [40]. The sensor monitors the changes in blood sugar in real time and pumps insulin as soon as a rise in blood sugar is detected, which greatly improves the quality of life of patients.

**Fig. 7.**
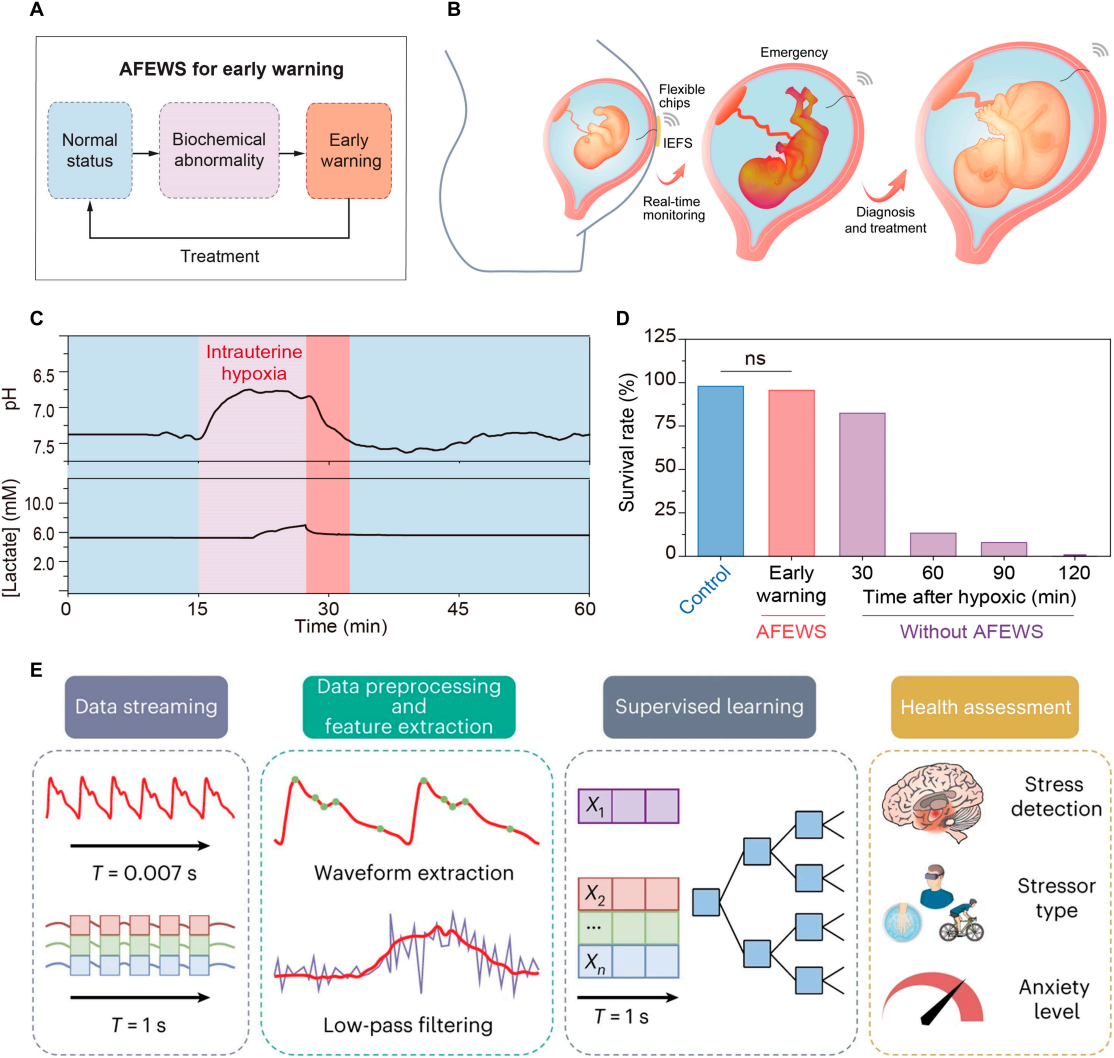
Early warning and diagnosis of diseases. (A) Block diagram shows how the IEFS works in AFEWS. (B) Schematic of the fiber sensor implanted in the uterus for real-time monitoring of biochemical signals during pregnancy. The signals monitored by the sensor were wirelessly transmitted through a flexible chip attached to the skin. (C) The pH and lactate concentration in the amniotic fluid of a rat under hypoxic conditions and after treatment were recorded by AFEWS. (D) The fetal rat’s survival rate of the pregnant rats under intrauterine hypoxia with or without warning from pregnancy to parturition. ns, no significant difference. (A to D) Reproduced with permission [18]. Copyright, 2024 Wiley-VCH. (E) Schematics of the machine learning architecture for data preprocessing, feature extraction, supervised learning, and evaluation. Reproduced with permission [86]. Copyright, 2024, Springer Nature.

1D implantable sensors also have great application potential in pregnancy disease monitoring. The traditional clinical evaluation of amniotic fluid has consisted primarily of B-mode ultrasonography and amniocentesis [85]. B-mode ultrasonography provides information related to amniotic fluid volume and fetal morphology but does not directly reveal biochemicals. Amniocentesis allows analysis of the extracted amniotic fluid, but the biochemical information obtained by amniocentesis is not real-time. As shown in Fig. 7A, by integrating an interface-stabilized electrochemical fiber sensor (IEFS) with a flexible wireless transmission patch, an amniotic fluid early warning system (AFEWS) was proposed for the first time [18]. This system was used for real-time monitoring of amniotic fluid biochemistry and early warning of pregnancy-related diseases. Specifically, the sensing end of the IEFS was immersed in amniotic fluid, and the other end was connected to a commercially available flexible chip on the skin. The detected biochemical information was converted into an electrical signal by the IEFS, which was collected and processed by the flexible chip and then wirelessly transmitted via Bluetooth module to a mobile terminal such as a cell phone or laptop computer (Fig. 7B).

Seven days after implantation, the accuracy of IEFS remained at 98%, demonstrating the sensor’s ability to accurately and stably monitor from mid to late gestation in pregnant rats. In addition, the sensor did not cause significant inflammation in pregnant rats according to hematoxylin–eosin staining, and immunofluorescence analysis results during implantation, verifying the feasibility of safe application to pregnant rats. It was then applied to the early warning of diseases such as intrauterine hypoxia, intrauterine infection, and fetal growth restriction. As shown in Fig. 7C, during intrauterine hypoxia, the pH value rapidly dropped from 7.4 to 6.7, and the lactate concentration increased from 5.2 to 7.2 mM. The AFEWS system provided timely warnings, and with external intervention, the fetal survival rate increased to 95% (Fig. 7D). The sensors provided a valuable window of time for timely intervention.

In addition to the standalone application of 1D sensors, they can also be integrated with existing medical devices. For example, medical catheters are widely used for drainage, but infections often occur at the catheter–tissue interface and cause local temperature increases at the implant site. There is usually a lag in measuring body temperature, which cannot reflect the local temperature change in time. By coating the hydrogel temperature sensor onto the surface of the medical catheter, it will be possible to monitor temperature changes caused by local infections in time [25]. In the brain infection model, the hydrogel-coated catheter was able to detect a change of 1 °C 3 h earlier than body temperature monitoring. After timely intervention, the survival rate of rats was as high as 90%, which was significantly higher than that of the group with monitoring body temperature (60%) and the group without intervention (50%). Moreover, the accuracy of the hydrogel temperature sensor remained stable within a single drain implant cycle of 7 d. During implantation, there was no obvious inflammatory response observed at the catheter with the brain, abdominal cavity, and bladder according to the results of Masson, hematoxylin–eosin staining, and immunofluorescence analysis, meeting the needs of stability and biocompatibility within a single drainage implantation cycle.

With the continuous development and application of implantable biosensors, the biochemical and physiological information collected by various sensors in real time will form massive amounts of data. Real-time analysis and feedback of these data will be of great value in assessing human health and diagnosing diseases. Artificial intelligence (AI) systems using machine learning techniques instantly analyze the vast data collected by sensors. By deeply analyzing data from healthy populations and patient groups, they recognize subtle abnormal changes that signal early warnings of disease (Fig. 7E) [86]. In addition, by training models on the massive amounts of data collected by sensors, machine learning programs can provide real-time disease diagnosis and give appropriate medication guidance and emergency measures based on the diagnosis [87].

Common machine learning algorithms include support vector machines (SVM), artificial neural networks, and convolutional neural networks [88–90]. SVM is suitable for handling datasets with relatively small sample sizes but high dimensions, such as electrocardiograms and electroencephalograms. Artificial neural network is suitable for medium-sized datasets, particularly for continuous signals like temperature, pressure, and chemical data. Convolutional neural network is particularly well suited for processing image and spatial data, such as ultrasound imaging, endoscopic images, and data with spatial and temporal dimensions. For example, by integrating defibrillators and pacemakers into 1D implantable sensors, the SVM model was able to precisely control electrical stimulation interventions in real time when abnormalities such as ventricular fibrillation and tachycardia were detected [78]. This effectively terminated lethal ventricular fibrillation and sustained ventricular tachycardia. The application of this solution will effectively address the randomness of heart disease onset and the short treatment window period, thereby reducing the mortality rate of heart disease.

## Challenges and Outlook

In recent years, advancements in materials and manufacturing technologies have significantly propelled the development of 1D implantable sensors within the biomedical field. Despite their immense potential for revolutionizing health monitoring, 1D implantable sensors are still in the nascent stages of development and encounter several pivotal challenges.

The stability of sensors is a critical concern that demands attention. The body environment is intricate and variable, exposing sensors to potential issues such as mechanical fatigue, chemical corrosion, and biodegradation during prolonged operation. Ensuring stability enables reliable long-term utilization of sensors within this dynamic internal milieu. In addition to environmental factors, the time scale is another dimension that needs to be considered. In the case of biochemical sensors, the current maximum operational duration for in vivo biochemical sensors stands at 6 months [80]. Considering the application requirements for chronic diseases like diabetes and cardiovascular diseases, measures must be taken to enhance the stability of sensitive materials employed in these sensors. This can be achieved by developing artificial recognition elements such as nanozymes and molecularly imprinted polymers to replace natural enzymes, antibodies, and aptamers. For instances where direct contact between analytes and sensors is unnecessary (e.g., mechanical and temperature sensors), more feasible approaches involve applying protective layers on sensitive materials through techniques like surface coatings or comprehensive encapsulation treatments.

Multifunctional integration is an important direction for the development of future 1D implantable sensors. Currently, the number of monitoring channels in sensors is relatively small, usually not exceeding 10 [15,18,27]. However, due to the intricate and diverse biological processes within the human body, comprehensive analysis of multiple signals is often required. Hence, integrating various sensing functions into a single sensor can provide more comprehensive and accurate health monitoring information. It is worth noting that this integration not only involves increasing the number of similar sensors but also includes the integration of different types of sensors. For instance, techniques like photolithography and micro-nano fabrication can further downsize the size of fiber electrodes. Different twisting methods can enhance the integration density of 1D sensors. Additionally, 3D printing technology allows for printing sensor arrays in various regions of the same electrode, thereby augmenting the number of sensors.

With the development of AI and big data technology, the data processing capabilities of 1D implantable sensors will be greatly enhanced. By integrating sensor data with intelligent algorithms, real-time analysis and prediction of biological signals become possible, offering new prospects for personalized medicine and precise diagnosis. For instance, machine learning-based algorithms analyze extensive biological signal data to detect potential health risks and disease trends, enabling early warning and intervention. However, the current algorithm design for 1D implantable sensors is relatively underdeveloped, primarily relying on general deep learning models such as supervised learning. While these models contribute to data postprocessing and scientific research to some extent, they still pose significant security risks [91]. Therefore, it is imperative to further improve their safety and accuracy to minimize the probability of misdiagnosis.

In summary, 1D implantable sensors hold immense potential for development in the fields of medicine and biotechnology. By optimizing materials stability, integrating multifunctional sensing technologies, and along with the combination of AI, 1D implantable sensors are set to become crucial for personalized and precision medicine. In the future, these sensors may become as indispensable to the human body as hair, safeguarding our health.
